# Evaluating the relationship between letermovir prophylaxis and medication regimen complexity Index over time in allogeneic hematopoietic stem cell transplant patients

**DOI:** 10.1177/10781552251330276

**Published:** 2025-05-05

**Authors:** Anthony Quach, Nimish Patel, Hailey Hirata, Annie Bui, Julie Trinh, Shreya Bahl, Ila M Saunders

**Affiliations:** 1 Department of Pharmacy, 21814 University of California, San Diego Health (UCSDH), San Diego, CA, USA; 2 Skaggs School of Pharmacy and Pharmaceutical Sciences, UC San Diego, La Jolla, CA, USA; 3 Department of Pharmacy, John Hopkins Medicine, Baltimore, MD, USA; 4 Department of Pharmacy, Sentara Norfolk General Hospital, Norfolk, VA, USA; 5 Department of Pharmacy, University of California, San Francisco (UCSF) Health, San Francisco, CA, USA

**Keywords:** Allogeneic hematopoietic stem cell transplant, cytomegalovirus, preemptive therapy, letermovir, prophylaxis, medication regimen complexity index, MRCI

## Abstract

**Background:**

The medication regimen complexity index (MRCI) quantifies patient-level regimen complexity, and higher scores are associated with adverse clinical outcomes. Characterization of regimens using the MRCI for allogeneic hematopoietic cell transplant (allo-HCT) recipients remains unexplored. Regimens may include letermovir which is used for cytomegalovirus prophylaxis and may prevent the need for addition of complex preemptive therapies. However, quantification of complexity in patients receiving letermovir has not been described.

**Objective:**

This study aimed to compare MRCI scores over a one-year period in allo-HCT recipients who received letermovir prophylaxis versus those who did not.

**Methods:**

A retrospective analysis included adults who underwent allo-HCT from January 1, 2016 to October 31, 2021. MRCI scores were calculated at admission, discharge, day +100, 6 months, and 1-year post-transplant.

**Results:**

A total of 218 patients were included, with 67 receiving letermovir and 151 not receiving letermovir. Median MRCI scores were comparable at discharge post allo-HCT (23 [10–39] vs 22 [12–37], *p *= 0.97). However, at day +100, patients in the letermovir group exhibited significantly higher median scores compared to the non-letermovir group (59 [46–74] vs 50 [37–67], *p *= 0.009). By 1-year post allo-HCT, no significant difference in scores was observed between groups (47 [30–68] vs 41 [27–61], *p *= 0.12).

**Conclusion and Relevance:**

This study revealed increased MRCI scores up to one year after transplantation in allo-HCT recipients receiving letermovir. The nonrandomized study design and potential patient differences between groups complicate the interpretation of the findings. Future analyses should aim to account for these differences.

## Introduction

The Medication Regimen Complexity Index (MRCI) is a useful tool to characterize the intricacy of medication regimens in populations where medication burden is considered high.^1^ The MRCI tool was created by George et al. in 2004 and has been used to evaluate medication regimen complexity in several patient populations, including patients diagnosed with chronic obstructive pulmonary disease, heart failure, various psychiatric conditions, and patients requiring heart transplant.^2–5^ The MRCI takes into account factors such as the number of medications, dosage forms, frequency of dosing, and additional administration instructions to generate a score summarizing the complexity of a patient's medication regimen.^1^ Higher MRCI scores indicate increased complexity and have been associated with increased medication nonadherence, hospital readmissions, and health resource utilization; these are clinical outcomes that contribute to patient safety and economic burden.^6–8^

Despite its applicability in quantifying medication regimen complexity in different patient populations, the MRCI has not yet been evaluated in allogeneic hematopoietic stem cell transplant (allo-HCT) recipients. Approximately nine thousand patients per year receive allo-HCT for the treatment of select non-malignant and malignant hematological diseases in the United States.^9^ Following hospital admission for HCT, allo-HCT recipients are typically discharged with multiple newly prescribed medications, including immunosuppressants, antimicrobials for infection prophylaxis, and supportive care to prevent post-transplant complications.^10,11^ The medication needs of the allo-HCT patient population are dynamic throughout the first year post-transplant, which often leads to increased non-adherence over time.^10,11^ Adherence within this population ranges from 33.0% to 94.7% across studies.^10^

A number of studies have shown that poor medication adherence in allo-HCT recipients are associated with significant sequelae, such as graft-versus-host disease (GVHD) and infections, both of which can result in considerable morbidity and mortality.^11–13^ Cytomegalovirus (CMV) infection, in particular, is prevalent in allo-HCT recipients due to the reactivation of latent virus caused by immunosuppression.^14^ If left untreated, CMV reactivation can progress to CMV disease, which can cause tissue damage in various organs.^14,15^ The management of CMV reactivation involves monitoring the viral load and initiating preemptive anti-viral therapy. The United States Food and Drug Administration (FDA) approved letermovir, a CMV DNA terminase complex inhibitor, for CMV infection prophylaxis in 2017.^16^ Initial clinical trials and studies demonstrated that letermovir is effective in reducing the incidence of CMV reactivation compared to a preemptive therapy approach in allo-HCT recipients.^17–19^

Given the limited research on medication regimen complexity in allo-HCT recipients and the potential benefits of letermovir in reducing CMV reactivation, this study aimed to evaluate the impact of letermovir prophylaxis on MRCI scores over a one-year time horizon in allo-HCT recipients. Our hypothesis was that letermovir prophylaxis would be associated with lower MRCI scores over time by reducing the need for preemptive therapy, thus alleviating the medication burden. Understanding this relationship is important for optimizing pharmacotherapy and potentially improving outcomes for allo-HCT recipients.

## Methods

### Study design and population

A retrospective cohort study was conducted among adult allo-HCT recipients at the University of California, San Diego Health (UCSDH) from January 1, 2016 to October 31, 2021. Inclusion criteria included patients who were age ≥ 18 years old, underwent allo-HCT at UCSDH, and survived 1 year post − HCT. Patients who had undergone more than one allo-HCT in the study time frame were excluded from the study. This study was approved by the Institutional Review Board (IRB) at UC San Diego (IRB# 191522) prior to data collection.

### Data collection

Trained reviewers extracted data from patients’ electronic health records, including demographics, clinical features of allo-HCT and risk of CMV disease, comorbidities, and medication lists. Demographic variables such as age, sex, and race were collected. Clinical features of allo-HCT included CMV serostatus, the primary reason for HCT, graft source, transplant type, conditioning regimen type, immunosuppression used, and pre-HCT Sorror comorbidity score. Patients were considered at high risk for CMV disease if they met one or more of the following criteria: had a mismatched unrelated/related donor or haploidentical donor, used umbilical cord blood as a stem cell source, underwent *ex vivo* T-cell depletion, or had acute GVHD of grade two or above.^20^ The pre-HCT Sorror comorbidity score evaluates the burden of comorbidities and predicts the prognosis of patients undergoing allo-HCT.^21^ Higher comorbidity scores have been associated with increased risk of complications and reduced overall survival post-transplant.^21^

Medication lists were extracted for each patient at five different time points: admission, discharge, day +100, 6 months, and 1-year post-transplant. Number of medications, dosage formulations, dosage frequency, and additional instructions were entered into an online MRCI tool (created and managed on REDCap^©^ hosted at UCSDH) to generate a MRCI score for each patient at the specified time point.^22,23^ Data were compared between patients who received letermovir versus patients who did not.

### Data analyses

Descriptive statistics were used to express clinical/demographic data, presented as number (%), mean ± standard deviation, or median (interquartile range). Categorical data were compared between groups using the chi-square or Fisher's exact test. Median total number of medications and MRCI scores were analyzed as continuous variables at each time point and compared between patients who received letermovir and patients who did not, using the Mann-Whitney U test. A p-value of <0.05 was considered statistically significant. Assuming a type I error rate of 5% and power of 80%, a total of 212 individuals would have been needed to detect a 5-unit difference in MRCI values between groups.

## Results

### Bivariate analyses

Among the 218 included patients, 67 received letermovir and the remainder did not receive letermovir. Across both groups, there were no significant differences between age (50.4 ± 15.6 vs 50.2 ± 15.5 years), male sex (55.2% vs. 58.9%), or race (53.7% vs 57.0% white). There were significant differences in CMV serostatus (*p *< 0.001), with a higher proportion of D-/R + individuals (29.9% vs 7.3%) among those who received letermovir versus those who did not. Additionally, the letermovir group had a significantly higher proportion of patients who were high risk for CMV disease (61.2% vs 27.2%, *p *< 0.001). Transplant type also differed significantly (*p *< 0.001), with a higher proportion of haploidentical transplants (40.3% vs 15.9%) in the letermovir group and a higher proportion of matched related donor transplants (7.5% vs 33.1%) in the non-letermovir group. Pre-HCT Sorror comorbidity scores were found to be significantly higher in the group that did not receive letermovir (1.66 ± 1.49 vs 2.67 ± 1.93, *p *< 0.001) (Table 1).

**Table 1.** Bivariate relationship between Clinical/Demographic characteristics and treatment group.

Covariate	Received Letermovir (n = 67)	Did Not Receive Letermovir (n = 151)	*p*-value
Age, mean ± SD	50.4 ± 15.6	50.2 ± 15.5	0.93
Sex, male n (%)	37 (55.2)	89 (58.9)	0.61
Race, n (%)			0.07
• Asian/Pacific Islander	10 (14.9)	15 (9.9)	
• Black	2 (3.0)	4 (2.6)	
• Hispanic	12 (17.9)	42 (27.8)	
• White	36 (53.7)	86 (57.0)	
• Other	7 (10.4)	4 (2.6)	
CMV serostatus, n (%)			<0.001
• D-/R-	2 (3.0)	42 (27.8)	
• D-/R+	20 (29.9)	11 (7.3)	
• D+/R-	14 (20.9)	29 (19.2)	
• D+/R+	31 (46.3)	69 (45.7)	
Primary reason for HCT, n (%)			0.51
• Acute Myeloid Leukemia	22 (32.8)	59 (39.1)	
• Myelodysplastic Syndrome	10 (14.9)	25 (16.6)	
• Non-Hodgkin's lymphoma	11 (16.4)	15 (9.9)	
• Acute lymphocytic leukemia	9 (13.4)	26 (17.2)	
• Other disease*	15 (22.4)	26 (17.2)	
Graft Source, n (%)			0.02
• Peripheral Blood Stem Cell	62 (92.5)	125 (82.8)	
• Cord blood	2 (3.0)	1 (0.7)	
• Bone Marrow	3 (4.5)	25 (16.6)	
Transplant Type, n (%)			<0.001
• Haplo	27 (40.3)	24 (15.9)	
• MMUD	2 (3.0)	5 (3.3)	
• MRD	5 (7.5)	50 (33.1)	
• MUD	33 (49.3)	72 (47.7)	
Conditioning Regimen, n (%)			0.64
• Myeloablative	35 (52.2)	84 (55.6)	
• Non-myeloablative/RIC	32 (47.8)	67 (44.4)	
Alemtuzumab Use Within 12 Months Prior to HCT, n (%)	1 (1.5)	0 (0)	0.31
Immunosuppression, n (%)			
• Cyclosporine	2 (3.0)	2 (1.3)	0.59
• Tacrolimus	60 (89.6)	145 (96.0)	0.06
• Mycophenolate**	44 (65.7)	30 (19.9)	<0.001
Acute GVHD (grade II or higher), n (%)	22 (32.8)	45 (29.8)	0.65
Risk for CMV Disease, n (%)			
• High Risk***	41 (61.2)	41 (27.2)	<0.001
ATG in conditioning regimen, n (%)	4 (6.0)	5 (3.3)	0.46
Disease status at HCT, n (%)			
• Remission	48 (71.6)	109 (72.2)	0.93
Pre-HCT Sorror comorbidity score, mean ± SD	1.66 ± 1.49	2.67 ± 1.93	<0.001

ATG: antithymocyte globulin; CMV: cytomegalovirus; GVHD: graft-versus-host-disease; Haplo: haploidentical; HCT: hematopoietic cell transplant; MMUD: mismatched unrelated donor; MRD: matched related donor; MUD: matched unrelated donor; RIC: reduced intensity conditioning.

*Other conditions requiring HCT: CML, HD, MM, SAA, myelofibrosis, blastic plasmacytoid dendritic cell neoplasm, plasma cell leukemia, acute undifferentiated leukemia, prolymphocytic leukemia, atypical CML

**Mycophenolate included mycophenolate mofetil, mycophenolate sodium, and mycophenolic acid.

***High risk: Patients having > 1 of the following criteria:

1. Mismatched unrelated/related donor or haploidentical donor

2. Underwent *ex-vivo* T-cell depletion within 12 months prior to HCT

3. Use of umbilical cord blood as stem cell source

5. Had acute GVHD grade II or above following HCT

Patients were classified as low risk if they did not meet the criteria for high risk.

### Total number of medications and medication regimen complexity

Total number of medications between the letermovir group and the non-letermovir group at each time point are shown in Table 2. The median number of medications from post-transplant through one year in those who received letermovir was 15.8, while for those who did not receive letermovir it was 14.3. The median (interquartile range, IQR) number of medications were similar between the letermovir and non-letermovir groups at admission (8 [4–11] vs 7 [4–11], *p *= 0.67). However, by day +100, the letermovir group had a significantly greater median number of medications than the non-letermovir group (18 [14–22] vs 15 [12–19], *p *= 0.008). This difference was transient, as by 1-year post-transplant, the median number of medications was similar again between the two groups (14 [10–19] vs 13 [8–18], *p *= 0.15).

**Table 2.** Comparison of total number of medications over one year between patients who received letermovir versus patients who did not receive letermovir.

Total # of Medications, Median [IQR]	Received Letermovir (n = 67)	Did Not Receive Letermovir (n = 151)	*p-*value
Admission	8 [4–11]	7 [4–11]	0.67
Discharge	15 [13–18]	14 [12–17]	0.05
Day +100	18 [14–22]	15 [12–19]	0.008
6 months post-HCT	16 [12–22]	15 [11–20]	0.32
1-year post-HCT	14 [10–19]	13 [8–18]	0.15

IQR: interquartile range.

MRCI scores between the two groups at serial time points are shown in Table 3. The average median MRCI value post-transplant through one year for the letermovir group was 51.3, while for the non-letermovir group it was 47.0. Similar to the median total number of medications, patients in the letermovir group had significantly higher median MRCI values compared to the non-letermovir group at day +100 (59 [46–74] vs 50 [37–67], *p *= 0.009). However, at 1-year post-HCT, the two groups had similar MRCI scores (47 [30–68] vs 41 [27–61], *p *= 0.12).

**Table 3.** Comparison of MRCI scores over one year between patients who received letermovir versus patients who did not receive letermovir.

MRCI Scores, Median [IQR]	Received Letermovir (n = 67)	Did Not Receive Letermovir (n = 151)	*p-*value
Admission	23 [10–39]	22 [12–37]	0.97
Discharge	49 [42–61]	47 [39–55]	0.06
Day +100	59 [46–74]	50 [37–67]	0.009
6 months post-HCT	50 [37–76]	50 [33–67]	0.20
1-year post-HCT	47 [30–68]	41 [27–61]	0.12

IQR: interquartile range.

Among individuals who were deemed to be high risk for CMV disease, there was no significant difference in the median MRCI values at any of the timepoints. Among individuals who were not high risk, the only significant differences in MRCI values between letermovir and non-letermovir recipients were at discharge and day +100.

## Discussion

To our knowledge, this is the first study to assess the MRCI specifically in allo-HCT recipients. Our findings align with previous research and confirms the significant prevalence of polypharmacy in this patient population.^10,11^ In contrast to most MRCI studies conducted on other patient groups that solely evaluated MRCI at a single time point, our investigation involved evaluating MRCI at multiple time points throughout the one-year post-transplant period. The only other study we are aware of that evaluated MRCI over time was a 2016 paper by Bryant et al., which aimed to quantify MRCI over a five-year time period following primary heart transplantation.^5^ Besides the difference in patient populations, our study parallels the Bryant et al. paper in that both our studies evaluated MRCI at serial time points following discharge from transplant. Heart transplant recipients were found to have persistently elevated medication counts and MRCI scores over time. It is worth noting that both study groups experienced the highest median total number of medications and MRCI scores at the Day +100 time point, which emphasizes the potentially heightened medication requirements during this specific period following allo-HCT. Overall, our study illustrates the dynamic nature of medication needs among allo-HCT recipients within the first-year post-transplant.

When comparing our MRCI findings to other patient populations, it becomes apparent that allo-HCT recipients require incredibly complex medication regimens. For instance, one study evaluating MRCI in patients with advanced chronic conditions who required palliative care reported a mean score of 38 (±16.54).^24^ A cohort of end-stage renal disease patients had a mean MRCI score of 42.3 (±11.9) in the first month after kidney transplant.^25^ Patient populations such as older adults with heart failure (32.1 ± 14.4), adult patients with HIV (21.8 ± 12.5), and adult patients with diabetes (23.0 ± 11.6) had lower mean MRCI scores.^26^ In contrast, median MRCI scores immediately after allo-HCT in both study groups exceeded 45. This further underscores the substantial medication burden faced by allo-HCT recipients.

Since our study did not assess medication adherence, it is challenging to determine the clinical implications of these MRCI scores or their association with non-adherence. However, many other studies show an association between MRCI and increased non-adherence leading to poorer clinical outcomes.^6,27,28^ Specifically in the post-HCT population, there is evidence linking medication non-adherence to increased rates of GVHD and infection.^11–13^ Pharmacists can play a crucial role in reducing medication burden for these patients through their involvement in medication management and transitions of care. Existing evidence already supports the value of pharmacists as part of a multidisciplinary HCT team,^29^ and incorporating MRCI as an additional measure can further validate their importance. This study provides valuable insights into medication complexity among allo-HCT recipients, highlighting the need for further research to elucidate the significance of MRCI in this patient population.

We hypothesized that the introduction of letermovir prophylaxis would reduce medication regimen complexity over time for allo-HCT recipients compared to just a preemptive therapy approach alone. Interestingly, our results refuted this hypothesis, revealing that patients who received letermovir had significantly higher number of medications and MRCI scores than those who did not receive letermovir at Day +100. However, by the end of the first year, there was no significant difference in the MRCI between the two groups. Several factors may have contributed to these outcomes, including potential variations in medication reconciliation capture rates between the groups and a higher proportion of patients at high risk for CMV disease in the letermovir group. Future analyses should consider these factors to gain a clearer understanding of the impact of letermovir prophylaxis on medication regimen complexity.

While this study offers valuable insights, it is important to acknowledge its limitations when interpreting these data. First, the nonrandomized nature of our study design may have introduced potential biases, particularly due to differences in baseline characteristics between the letermovir and preemptive therapy groups. This may have contributed to the observed differences in MRCI scores, especially at Day +100. Significant differences in MRCI values between letermovir and non-letermovir recipients may also be dependent upon the distribution of CMV donor/recipient types in a population. Our distribution of donor/recipient types may differ from other institutions which also limits the external validity of the findings. Second, the retrospective evaluation of medication lists from electronic medical records prevented the assessment of medication adherence and the recording of additional instructions received in the outpatient setting. As a result, the relationship between MRCI and adherence in this patient population was not evaluated and should be considered in future studies. Third, we included patients from only one academic health system which may not fully represent the typical medication regimens and prescribing patterns in other health systems for this patient population. Lastly, we did not evaluate the relationship between MRCI and clinical outcomes. Further studies are warranted to evaluate the relationship between medication regimen complexity, medication adherence, and clinical outcomes in allo-HCT recipients.

## Conclusion and relevance

In conclusion, our study confirms a high medication burden among allo-HCT recipients, with notable increases in MRCI scores from admission to discharge and throughout the first-year post-transplant. These findings emphasize the need for proactive interventions to reduce medication regimen complexity in this vulnerable patient population. Contrary to our hypothesis, the use of letermovir prophylaxis did not result in a decrease in medication regimen complexity over time. Future research considering potential confounding factors is needed to fully understand its impact. Our findings lay the groundwork for future investigations into the relationship between MRCI and clinical outcomes in this population.
